# Investigating the Impact of AI on Shared Decision-Making in Post-Kidney Transplant Care (PRIMA-AI): Protocol for a Randomized Controlled Trial

**DOI:** 10.2196/54857

**Published:** 2024-04-01

**Authors:** Bilgin Osmanodja, Zeineb Sassi, Sascha Eickmann, Carla Maria Hansen, Roland Roller, Aljoscha Burchardt, David Samhammer, Peter Dabrock, Sebastian Möller, Klemens Budde, Anne Herrmann

**Affiliations:** 1 Department of Nephrology and Medical Intensive Care Charité – Universitätsmedizin Berlin Berlin Germany; 2 Department of Epidemiology and Preventive Medicine Medical Sociology University Regensburg Regensburg Germany; 3 German Research Center for Artificial Intelligence Berlin Germany; 4 Institute for Systematic Theology II (Ethics) Friedrich-Alexander University Erlangen Nürnberg Erlangen Germany; 5 Quality and Usability Lab Technical University of Berlin Berlin Germany; 6 School of Medicine and Public Health University of Newcastle Callaghan Australia

**Keywords:** shared decision-making, SDM, kidney transplantation, artificial intelligence, AI, decision-support system, DSS, qualitative research

## Abstract

**Background:**

Patients after kidney transplantation eventually face the risk of graft loss with the concomitant need for dialysis or retransplantation. Choosing the right kidney replacement therapy after graft loss is an important preference-sensitive decision for kidney transplant recipients. However, the rate of conversations about treatment options after kidney graft loss has been shown to be as low as 13% in previous studies. It is unknown whether the implementation of artificial intelligence (AI)–based risk prediction models can increase the number of conversations about treatment options after graft loss and how this might influence the associated shared decision-making (SDM).

**Objective:**

This study aims to explore the impact of AI-based risk prediction for the risk of graft loss on the frequency of conversations about the treatment options after graft loss, as well as the associated SDM process.

**Methods:**

This is a 2-year, prospective, randomized, 2-armed, parallel-group, single-center trial in a German kidney transplant center. All patients will receive the same routine post–kidney transplant care that usually includes follow-up visits every 3 months at the kidney transplant center. For patients in the intervention arm, physicians will be assisted by a validated and previously published AI-based risk prediction system that estimates the risk for graft loss in the next year, starting from 3 months after randomization until 24 months after randomization. The study population will consist of 122 kidney transplant recipients >12 months after transplantation, who are at least 18 years of age, are able to communicate in German, and have an estimated glomerular filtration rate <30 mL/min/1.73 m^2^. Patients with multi-organ transplantation, or who are not able to communicate in German, as well as underage patients, cannot participate. For the primary end point, the proportion of patients who have had a conversation about their treatment options after graft loss is compared at 12 months after randomization. Additionally, 2 different assessment tools for SDM, the CollaboRATE mean score and the Control Preference Scale, are compared between the 2 groups at 12 months and 24 months after randomization. Furthermore, recordings of patient-physician conversations, as well as semistructured interviews with patients, support persons, and physicians, are performed to support the quantitative results.

**Results:**

The enrollment for the study is ongoing. The first results are expected to be submitted for publication in 2025.

**Conclusions:**

This is the first study to examine the influence of AI-based risk prediction on physician-patient interaction in the context of kidney transplantation. We use a mixed methods approach by combining a randomized design with a simple quantitative end point (frequency of conversations), different quantitative measurements for SDM, and several qualitative research methods (eg, records of physician-patient conversations and semistructured interviews) to examine the implementation of AI-based risk prediction in the clinic.

**Trial Registration:**

ClinicalTrials.gov NCT06056518; https://clinicaltrials.gov/study/NCT06056518

**International Registered Report Identifier (IRRID):**

PRR1-10.2196/54857

## Introduction

Shared decision-making (SDM) is increasingly important in health policy and clinical practice. Over the past decades, researchers, patient advocates, and policymakers all over the world have increased efforts to shift health care from a paternalistic to a patient-centered approach, focusing on the patient as a person [1,2]. Kidney transplantation is the most frequently performed solid organ transplantation and although the application of SDM prior to kidney transplantation has been discussed, little is known about how to improve SDM after transplantation [3]. This is surprising as there are numerous potentially preference-sensitive decisions that patients, support persons, and physicians have to make in this setting, for instance relating to the management of comorbidities, effects of treatment on fertility, use of immunosuppressant drugs, and resulting second cancers [4,5].

In patients with chronic kidney disease, choosing the right kidney replacement therapy and the optimal timing for kidney replacement therapy is an important preference-sensitive decision. Implementing SDM has been shown to leave more patients satisfied with their choice of dialysis modality [6]. Not only the modality itself but also details like the vascular access and the specific dialysis regimen are preference-sensitive and should be part of an SDM process that also considers age-dependent needs [7-9].

Since most patients after kidney transplantation eventually face the risk of graft loss with the concomitant need for dialysis or retransplantation, comparable considerations should be applied to kidney transplant recipients at risk for graft loss. However, the rate of conversations about treatment options after kidney graft loss is as low as 13% in conventional physician-centered care settings [10]. This leaves room for optimization with respect to the frequency of conversations as well as the associated SDM process. While care-based interventions have shown to be effective in increasing the frequency of conversations about treatment options after graft loss, it is unknown whether the implementation of artificial intelligence (AI)–based risk prediction models can have such influence on physician-patient interactions [10].

Different risk prediction models for graft loss using methods of statistics or AI have been introduced and show good predictive performance [11,12]. More importantly, they are more accurate than experienced physicians in predicting risks for graft loss [11,13]. Implementing such models into routine care could indicate patients at risk for graft loss and thereby increase the frequency of conversations about treatment options after graft loss. However, little is known about the impact of AI-based interventions on the interaction between patients, support persons, and physicians [14].

While there are several studies discussing the potential of AI to support SDM in the bioethical literature [15], there is a lack of empirical studies to systematically investigate the impact of AI on SDM [16].

In this study, we aim to evaluate the influence of an AI-based risk prediction model for 1-year risk of graft loss on the frequency of conversations about treatment options after graft loss, as well as the associated SDM process, in patients who had kidney transplants with low estimated glomerular filtration rate (eGFR).

## Methods

### Study Design and Setting

**Figure 1:**
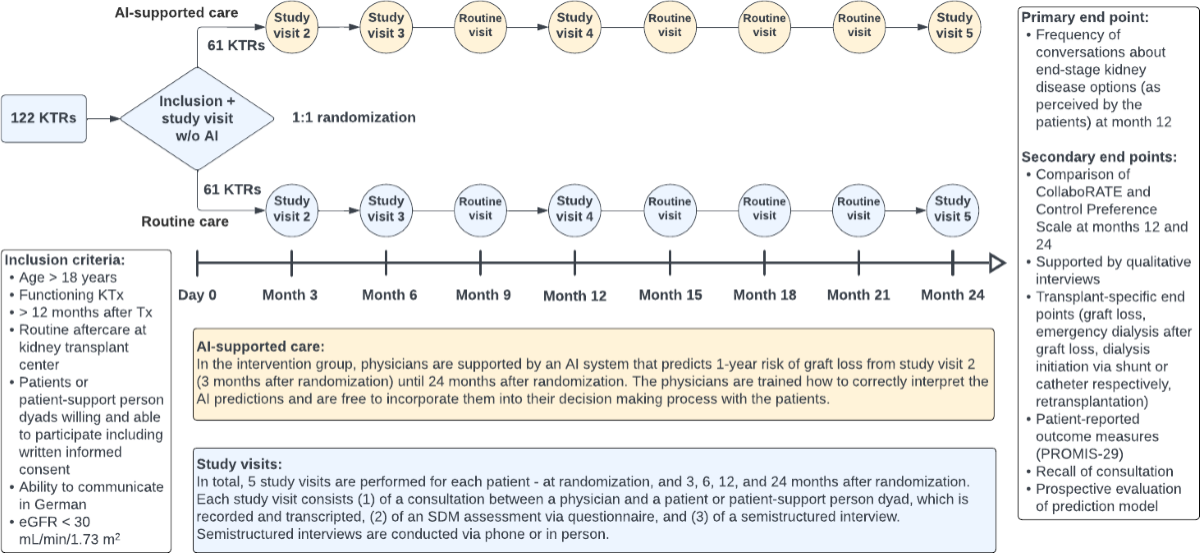
Study flowchart summarizing inclusion criteria, interventions, and end points. An estimated number of 122 KTRs will be recruited in the outpatient clinic of a tertiary care center and randomized 1:1 into routine care or AI-supported care and followed up over 24 months. AI: artificial intelligence; eGFR: estimated glomerular filtration rate; KTR: kidney transplant recipient; KTx: kidney transplantation; PROMIS-29: Patient Reported Outcomes Measurement Information System 29; SDM: shared decision-making; Tx: transplantation.

The Prospectively investigating the Impact of AI on Shared Decision-Making in Post-Kidney Transplant Care (PRIMA-AI) trial is a 2-year, prospective, randomized, 2-armed, parallel-group, single-center trial in a German kidney transplant center (KTC). Patients more than 12 months after kidney transplantation with eGFR <30 mL/min/1.73 m^2^ and support persons will be randomized to routine care or to AI-supported care in a 1:1 ratio. During the study, 5 study visits are planned at the KTC—at randomization, and 3, 6, 12, and 24 months after randomization (Figure 1). In both groups, routine care visits will be scheduled as needed, depending on the patient’s medical condition and time posttransplant, which is usually every 3 months.

### Recruitment Process

Patients after kidney transplantation regularly undergo routine follow-up at the KTC in an outpatient clinic. Patient data are available in a proprietary electronic health record that allows data extraction for research purposes [17]. Patients who are potentially eligible for the study based on their previous medical data will be contacted via telephone 1 week before their next scheduled appointment and will be asked if they are interested in study participation. If patients are potentially interested, they are provided detailed information about the study by trained study physicians and provide written informed consent after checking inclusion and exclusion criteria at the time of their next outpatient visit at the KTC. Recruitment will occur in the regular outpatient treatment at the KTC by designated study physicians who are not the treating physicians. After recruitment and randomization, the first study visit is performed. No study investigations are performed before written informed consent is obtained from the patients. The KTC is a tertiary care center specializing in kidney transplantation, where approximately 200 kidney transplantations per year are performed.

### Participants

Participants may enter the trial if all the following apply: (1) they provide written informed consent, (2) they are patients with a functioning kidney allograft, (3) it has been at least 12 months after kidney transplantation, (4) they have an eGFR <30 mL/min/1.73 m^2^ according to Chronic Kidney Disease Epidemiology Collaboration (CKD-EPI) 2021 formula, (5) they are aged at least 18 years, (6) they can communicate in German, and (7) they attend regular follow-ups at KTC. Participants may not enter the trial if any of the following apply: (1) they have undergone multi-organ transplantation, (2) it has been less than 12 months after kidney transplantation, (3) they have an eGFR >30 mL/min/1.73 m^2^ according to CKD-EPI 2021 formula, (4) they are aged less than 18 years, (5) they cannot communicate in German, (6) they do not attend regular follow-ups at KTC, and (7) they enrolled in another interventional study less than 1 month before participation in this study. Additionally, support persons will be eligible if they are nominated by the patient as someone helping them cope with the consequences of their kidney transplant through support, encouragement, and communication. Support persons will be aged 18 years or older, German-speaking, and able to provide informed consent. Treating physicians who work at the participating clinics and use the AI-based decision-support system (DSS) will be eligible to participate in the study. Clinic staff will record the age and gender of nonconsenters who provided permission, which allows for the examination of consent bias.

### Sample Size

The primary analysis will be performed based on the frequency of conversations about treatment options after graft loss as perceived by the patient, which is assessed in a questionnaire after the respective consultation. Based on the data from Bissonnette et al [10], we estimate that in the control group, 10%-15% of patients will have a conversation about therapy options at the end of graft function. Regarding the intervention studied herein, we estimate that this frequency can be increased to 40%-45%, which is half of the effect size achieved by Bissonnette et al [10]. Using a 2-sided *χ*^2^ test, an α error of .05, and a power of 80%, 25-49 patients per group are needed. Estimating a dropout rate of 10% per year, this increases to 31-61 patients per group. Therefore, 61 patients per group were regarded as sufficient. In order to identify 122 participating patients, local study personnel will have to screen approximately 150 potentially eligible kidney transplant recipients over a period of 6 months.

### Randomization

Randomization will be performed using a predefined variable block randomization scheme using a web-based randomization service [18]. After screening for eligibility and assignment of the individual patient identifier, each patient-support person dyad can only be assigned once to 1 of the treatment arms. The sites will record the time of randomization.

### Blinding

Blinding of participants and study staff is not possible given the nature of the intervention.

### Intervention

#### Control Group: Routine Care

Routine care will be scheduled according to the current standard of care [19], which depends on the time after transplantation, medical condition, and other individual factors. Standard immunosuppression will be applied according to the international recommendations [20]. The prophylaxis and treatment of infections will follow the current standard of care as outlined in recent guidelines [21-26], for example, *Pneumocystis jirovecii* pneumonia prophylaxis for 4 to 6 months, cytomegalovirus prophylaxis according to the guidelines, and regular posttransplant BK virus monitoring according to guidelines [19]. Screenings for the antibodies against human leukocyte antigen once within the first 3 months, after 1 year, and every year thereafter, as well as in case of suspected rejection [27]. The overall medical treatment of kidney transplant recipients will be performed according to the Kidney Disease Improving Global Outcomes guidelines [19] and in patients with suspected rejection, a kidney biopsy should be performed and classified according to the most recent Banff criteria [28]. An integral part of the current routine aftercare is regular visits to the home nephrologist and KTC, where data are captured in an electronic health record [17]. Table 1 highlights the proposed schedule for posttransplant aftercare. The proposed schedule is adapted to the individual patient’s needs.

**Table 1:** Frequency of routine visits in the outpatient clinic of the kidney transplant center (KTC) depending on time after transplantation.

Time after transplantation	Frequency of routine visits
Month 1	Once per week after discharge at the KTC
Month 2	Once per week
Month 3	Once per 2 weeks
Months 4 to 6	Once per 3 weeks
Months 6 to 12	Once per month
After 12 months	Once every 3 months

#### Intervention Group: AI-Supported Care

The kidney transplant recipients, who are randomized to the interventional arm, will receive identical routine posttransplant care as patients in the control group (see Table 1). In addition, the physicians in the KTC will be provided an AI-based decision support system making predictions for the risk of graft loss based on the patient’s most recent clinical, laboratory, and histopathology data, beginning from the second visit. A previous version of the AI-based decision support system has been studied in silico and in a reader study [11]. The physicians are provided a recommendation on how to proceed in case of a high-risk score. Briefly, physicians are recommended to inform the patients about this risk and discuss the potential necessity of return to dialysis or retransplantation, as well as planning, which mode of renal replacement therapy is preferred by the patient (hemodialysis, peritoneal dialysis, or listing for retransplantation by living donation). Depending on the patient’s preference, preparation for a smooth transition to dialysis or retransplantation should be performed.

### Outcomes

#### Primary End Points

For the primary end point, the frequency of conversations about treatment options after graft loss as perceived by the patient will be compared between the 2 groups at 12 months after randomization.

#### Secondary End Points

As the main secondary end points, the 2 different assessment tools for SDM, the CollaboRATE mean score and the Control Preference Scale (CPS; adapted version as used in previous studies [29]), are compared between the 2 groups at 12 months after randomization. The CPS is widely used as a validated tool to assess involvement in SDM [29,30]. However, it has been suggested that CPS may be misleading in some contexts of medical decision-making since it does refer explicitly to a “decision,” whereas, CollaboRATE is a brief process-orientated measure that recognizes that study participants may not always realize that a decision has been made or have difficulties focusing on 1 decision in the context of a complex care experience [31,32]. CollaboRATE has shown discriminative validity and interrater reliability. To supplement the quantitative outcome measures, qualitative data collection in the form of interviews will be performed and analyzed. All questionnaires and interview guides will be developed with the help of a multidisciplinary research group and pilot tested with patients and support persons to optimize acceptability and feasibility. Other secondary end points are summarized in Textbox 1.

**Secondary end points and time frame after which they are assessed**

**End points**

- CollaboRATE mean score of all study visits from study inclusion until month 12 (values 0-9, higher values indicating better outcome)
- CollaboRATE mean score of all study visits from study inclusion until month 24 (values 0-9, higher values indicating better outcome)
- Mean Control Preferences Scale of all study visits from study inclusion until month 12 (values 1-5, higher values indicating better outcome)
- Mean Control Preferences Scale of all study visits from study inclusion until month 24 (values 1-5, higher values indicating better outcome)
- Frequency of kidney replacement therapy after graft loss at 12 months
- Frequency of kidney replacement therapy after graft loss at 24 months
- Frequency of emergency dialysis after graft loss at 12 months
- Frequency of emergency dialysis after graft loss at 24 months
- Frequency of dialysis initiation via arteriovenous-shunt after graft loss at 12 months
- Frequency of dialysis initiation via arteriovenous-shunt after graft loss at 24 months
- Frequency of dialysis initiation via permanent catheter after graft loss at 12 months
- Frequency of dialysis initiation via permanent catheter after graft loss at 24 months
- Frequency of retransplantation after graft loss at 12 months
- Frequency of retransplantation after graft loss at 24 months
- Qualitative analysis of semistructured interviews after at 24 months
- Qualitative analysis of physician-patient conversations after at 24 months
- Frequency of conversations about treatment options after graft loss as perceived by the patient will be compared between the 2 groups at 24 months

### Procedures

#### Overview

The study plan is summarized in Table 2.

**Table 2:** Study plan including the data and time points at which these are assessed.

Data	Day 0 (visit 1)	Month 3 (visit 2)	Month 6 (visit 3)	Month 12 (visit 4)	Month 24 (visit 5)
Consent	✓				
Inclusion or exclusion criteria	✓				
Randomization	✓				
AI^a^ system (intervention group)		✓	✓	✓	✓
Demography, transplant data, medical history, and height	✓	✓	✓	✓	✓
CollaboRATE score	✓	✓	✓	✓	✓
Control preferences scale	✓	✓	✓	✓	✓
Qualitative interviews	✓	✓	✓	✓	✓
Recall	✓	✓	✓	✓	✓
Vital signs (BP^b^, HR^c^, and weight)	✓	✓	✓	✓	✓
Safety lab	✓			✓	✓
Serum creatinine and eGFR^d^	✓	✓	✓	✓	✓
Proteinuria or albuminuria	✓	✓	✓	✓	✓
HLA^e^ antibodies	✓			✓	
Tacrolimus trough level	✓	✓	✓	✓	✓
Quality of life (PROMIS-29^f^)	✓	✓	✓	✓	✓
Clinical assessment	✓	✓	✓	✓	✓
AEs^g^ or SAEs^h^, AEs of interest, graft loss, and death	✓	✓	✓	✓	✓
Hospitalizations (reason, length, and type)	✓	✓	✓	✓	✓
Physician visits and contacts	✓	✓	✓	✓	✓
Immunosuppression	✓	✓	✓	✓	✓
Prior or concomitant medications	✓	✓	✓	✓	✓

^a^AI: artificial intelligence.

^b^BP: blood pressure.

^c^HR: heart rate.

^d^eGFR: estimated glomerular filtration rate.

^e^HLA: human leukocyte antigen.

^f^PROMIS-29: Patient Reported Outcomes Measurement Information System 29.

^g^AE: adverse event.

^h^SAE: serious adverse event.

#### Details of the AI-Based DSS and Recommended Procedures

An AI-based prediction model will be implemented to provide real-time predictions of the 1-year risk for graft loss based on the patient’s clinical, laboratory, immunological, and histopathological data. A previous version of the prediction model has been described in detail before, and all substantial changes will be made publically available. The predictions are provided as regression scores from 0 to 100 with higher scores indicating higher risk. For ease of interpretability, each score is classified into low-, medium-, or high-risk categories, which are color-coded as green, yellow, and red. Along with the risk score and risk category over time, relevant risk factors are provided to increase interpretability for the latest risk assessment.

Physicians will undergo training, in which technical details of the underlying model (training cohort, inclusion and exclusion criteria, end point definition, and performance), and results of preclinical studies are presented [11]. Hereby, the physicians can gain familiarity with the AI-DSS and also discuss potential limitations with the developers and study physicians. These training sessions will be recorded and analyzed as part of the qualitative subproject.

During the study, the physicians are advised to use the AI-DSS as an additional source of information and be especially cautious in case of high risks detected by the AI-DSS. In any case, it will be left to the physician’s choice on how to include the system’s information in the conversation with patients and support persons.

However, if the AI-DSS shows a high risk for graft loss, the physicians are free to discuss this with the patient and offer 1 procedure depending on their own assessment. First, physician—high risk for graft loss: if the physician agrees that there is a high risk of graft loss in a patient during the next year, the physician is advised to discuss this risk with the patient, when the situation is appropriate (enough time, no other major problems to be discussed, etc) or to discuss it during the next appointment. The physician should assess, whether the preferred renal replacement modality (hemodialysis, peritoneal dialysis, and retransplantation) of the patient is known and take measures to ensure a smooth transition without the need for emergency hospitalization (shunt planning, peritoneal dialysis catheter implantation, appointment for living donation, etc). Second, physician—low risk for graft loss: if the physician disagrees with the AI system and estimates a low risk for graft loss in a particular patient, the physician is advised to reconsider potential risk factors. The physician is free to incorporate or ignore the AI-DSS assessment into the SDM with patients and support persons.

#### Details on Qualitative Research Methodology

##### Overview

The physician-patient conversation will be recorded if physicians, patients, and support persons do not withdraw consent at randomization, as well as 3, 6, 12, and 24 months after randomization. Additionally, patients will be interviewed at randomization, as well as 3, 6, 12, and 24 months after randomization. Data collection at randomization, as well as 12 and 24 months after randomization will be conducted face-to-face, if possible and preferred by study participants. Data collection at 3 and 6 months after randomization will be conducted via telephone. This is to reduce the research-related burden on the participants. It has been suggested that telephone interviews produce similar quality data as face-to-face interviews [33-35]. Also, patients may appreciate being interviewed via telephone as they may feel more relaxed when being interviewed on the telephone and may find it easier to rearrange a telephone interview, rather than having to rearrange a face-to-face interview [36-39]. Participants will be encouraged to tell their views on how the AI-based DSS impacted physician-patient-support person communication and how treatment decisions were made, in the way they prefer, with as little interruption as possible from the interviewer. This narrative approach will help elicit the variety and interplay of potential factors related to treatment discussions [40]. The initial narrative will be followed by semistructured questions which will be developed based on a literature review and discussions among the research team which involves experts in the areas of medicine, communication and behavioral science, health services research, ethics, and medical informatics.

##### Influence of AI-Based Decision Support on the Normative Foundations of the Use of AI in SDM

Participants will be asked questions on their perceptions of concepts such as trust, agency, or transparency, for instance about how they evaluate the outputs of the tool and how these outputs relate to their physicians’ judgments. Participants may also be asked to report on their views on the system’s validity, effectiveness, and the perceived likelihood of errors, as well as on who is morally and legally liable for single treatment decisions.

##### Use of AI-Based Decision Support and Barriers and Enablers to its Implementation

Participants will be asked about their perceptions of acceptability, ease of use, agreement with specific components of the system’s outputs, and self-efficacy (ie, the belief that one can understand and use the system’s output). Participants will also be asked about further potential barriers to the use of AI in routine care, such as environmental factors like time pressure. These questions will be informed by the literature and discussions among the research team [41,42].

##### Sociodemographic and Disease Variables

Sociodemographic and disease variables obtained from patients and support persons will include gender, marital status, country of birth, postcode, highest level of education completed, income, and perceived health status. The support persons will also be asked to self-report their relationship to the patient and whether they are living with the patient. All sociodemographic and disease variables will be assessed at baseline and follow-ups to account for changes in participants’ circumstances which may affect their views and experiences [43]. With patients’ permission, information regarding diagnosis, disease stage, and treatments received will be obtained from patients’ medical records to decrease the research-related burden on patients. At the end of the interview, participants will be given the option to provide additional comments.

### Statistical Analysis

Preliminary analysis will involve computing descriptive statistics for all quantitative variables. For continuous variables, means, SDs, and quartiles will be estimated, while categorical variables will be summarized with counts and percentages in each category. Summaries will be performed by group and by assessment, as well as for the entire study group. Primary data analysis of the primary end point will involve comparing the frequencies of conversations about treatment options after graft loss between both groups using a 2-sided *χ*^2^ test. Secondary data analysis will include a comparison in secondary outcomes between both groups using a *χ*^2^ test. In general, test results will be described as significant if their *P* values are less than .05 without adjustments for multiple inferences. Multiple imputations will be used to deal with missing data.

### Qualitative Research Analysis

Interviews will be transcribed verbatim. The interviews will be recorded for the purpose of transcription on provided devices and will be deleted after the end of the research process. Transcripts will be checked for accuracy by 1 researcher and analyzed using framework analysis. This approach belongs to a broad family of qualitative data analysis methods often related to as “thematic analysis” or “qualitative content analysis” [44]. As suggested by these approaches, both manifest and latent content will be analyzed and descriptive and explanatory conclusions will be drawn from the data [45]. Each interview will serve as a unit of analysis. A journal of reasoning and additional ideas regarding data analysis will help ensure transparency in the coding process. This strategy has been extensively used to facilitate the reconstruction of the analysis and provide justification for the analytical steps undertaken [46,47]. Codes will frequently be compared with each other and parts of the material will be recoded, if necessary, as an intercoder agreement test and additional measure for reliability [48]. This method of qualitative data analysis will provide a systematic model for mapping and interpreting the data and is thus considered appropriate to develop a profound in-depth understanding of participants’ communication experiences and preferences [46,49].

### Data Management

Designated investigator staff must enter the information required by the protocol into the assigned database. Individual interviews, telephone interviews, and patient-physician conversations will be audio recorded, transcribed verbatim by a professional transcriptionist or a research team member, deidentified, and filed for data management and analysis. All qualitative data will be collected by a research team member with extensive experience in qualitative research.

### Safety

Safety assessments will consist of monitoring and recording all adverse events (AEs) and serious AEs, the regular monitoring of blood chemistry and urine, and regular monitoring of vital signs, physical condition, and body weight. Appropriate sponsor personnel and investigators will monitor the safety of the participants throughout the conduct of the study. AEs mean “any untoward occurrence in a trial subject administered an investigational medicinal product and which does not necessarily have a causal relationship with this treatment” [50]. In the PRIMA-AI trial, subjects will not receive any investigational medicinal product, but physicians will be assisted by an AI-based decision support system. Thus, in this study, an AE can be any unfavorable and unintended sign, symptom, or disease (including concomitant illness), deterioration of a preexisting illness, accident, any suspected drug reaction, or a clinically relevant change of laboratory values whether or not considered to be related to the AI-based intervention. Adverse reactions (ARs) mean “all untoward and unintended responses to an investigational medicinal product unrelated to the dose administered” [50]. In PRIMA-AI, subjects will not receive any investigational medicinal product. Therefore, the definition of AR implies in this trial a reasonable possibility of a causal relationship between the event and the investigational AI-based intervention.

The period of observation for AEs extends from the baseline visit (month 0, visit 1) after informed consent was given until and including month 24 (visit 5). AEs have to be followed up until resolution or stabilization or until the subject’s end-of-study visit (month 24, visit 5), whichever comes first. Preexisting conditions that do not worsen during the course of the study are not reportable as AE. To determine whether a condition has worsened, it is compared to the condition of the subject at baseline visit (month 0, visit 1). All AEs, whether volunteered by the patient, discovered by investigator questioning, or detected through physical examination, laboratory test, or other means will be assessed and recorded in detail in the subject’s file and on the case report form, the AE report form. All AEs will be coded appropriately at the end of the clinical study using MeDRA or, in addition, on request by the study coordinator.

The following information must be recorded: (1) AE diagnosis (if possible) or main symptom; (2) date (and time, if relevant) of onset; (3) severity (maximum observed); (4) causal relationship (reasonable possibility or no reasonable possibility); (5) seriousness (yes or no); (6) outcome; (7) action taken with the study intervention; (8) AE leading to discontinuation of the study subject (yes or no); (9) treatment of AE; and (10) stop date (and time, if relevant). For the assessment of severity, the investigator should use grades from 1 to 5 as outlined in Textbox 2.

**Grades of assessments of severity for adverse events.**

**Grade 1**

Mild or asymptomatic symptoms, clinical or diagnostic observations only, and intervention not indicated.

**Grade 2**

Moderate, minimal, local, or noninvasive intervention indicated, and limiting age-appropriate instrumental activities of daily living. Instrumental activities of daily living are activities such as preparing meals, shopping for groceries or clothes, using the telephone, managing money.

**Grade 3**

Severe or medically significant but not immediately life-threatening, hospitalization or prolongation of hospitalization indicated, disabling, and limiting self-care activities of daily living. Self-care activities of daily living are activities such as bathing, dressing and undressing, feeding oneself, using the toilet, taking medications, and not being bedridden.

**Grade 4**

Life-threatening consequences and urgent intervention indicated.

**Grade 5**

Death related to adverse event.

### Ethical Considerations

Written informed consent will be collected from all study participants before performing any research activity by a research team member. To ensure the eligibility of a participate in the trial, the researcher will review the inclusion and exclusion criteria with potential participants prior to beginning the informed consent process. Prospective participants will be asked to personally sign and date the latest approved version of the informed consent forms before any trial procedure is performed. During the informed consent process, study participants will be informed of their right to withdraw from the study at any time without any impact on their treatment at the KTC. This study will be conducted in accordance with the tenets of the Declaration of Helsinki (1996). The ethics committee of Charité—Universitätsmedizin Berlin approved this study (EA1/177/23; August 08, 2023).

The trial results will be disseminated through a variety of strategies, including academic publications, research reports, infographics, media releases, and community events. Trial participants’ anonymity and confidentiality will be protected during these activities by removing all identifiable information from the knowledge dissemination products.

## Results

As of March 10, 2024, we enrolled 19 participants. The first results are expected to be submitted for publication in 2025.

## Discussion

### Principal Findings

This study is designed to investigate the influence of AI-based risk prediction on the frequency of conversations about treatment options after graft loss and the associated SDM process with a randomized design using a mixed methods approach. It is the first study in the context of kidney transplantation to analyze the potential effects of AI-based interventions on physician-patient interaction.

Despite tremendous enthusiasm surrounding the potential for AI to improve medical prognosis, diagnosis, and decision-making, only limited empirical data are available to help understand patients’ and physicians’ perspectives on how AI impacts their interactions, specifically SDM [16]. In the context of kidney transplantation, AI research has mostly focused on technical and medical challenges relating to robustness and implementation, excluding the sociotechnical environment into which such systems are embedded [12-14]. In the treatment of patients with cancer, an intervention that delivered machine learning mortality predictions with behavioral nudges to oncology clinicians significantly increased the rate of serious illness conversations [51,52]. While serious illness conversations are only in part comparable to conversations about graft loss, returning to dialysis after transplantation is also a life-changing event with serious effects on quality of life. We argue that increasing the frequency of conversation about treatment options after graft loss and improving SDM may improve patient satisfaction comparable to patients with chronic kidney disease without transplantation [6].

### Strengths and Limitations

The main strength of this study is the mixed methods approach that will support the analysis of the primary end point with validated assessment tools for SDM and qualitative data from semistructured interviews. It is the first study in the context of kidney transplantation that investigates the impact of AI-based risk prediction on physician-patient interaction. While performed in a tertiary care setting, transplant nephrologists often serve as primary caregivers to kidney transplant recipients. Therefore, the results may generalize to a broader range of care settings as well. The randomized design enables to detect potential effects of the AI-based risk prediction on the conversation frequency about graft loss, which can improve patient information and patient care. It also provides us with a control group, in which SDM about treatment options after graft loss in kidney transplantation can be studied without any intervention, which has not been done so far. Another strength is the long observation time of 24 months, which enables to detect potential adaptations of physicians, patients, and support persons to the novel AI system and the emergence of a sociotechnical system in the recordings of patient-physician interactions as well as the semistructured interviews.

The main limitation is that potential effects of the AI intervention on SDM are indirect since no specific SDM intervention has been implemented and the AI system is not designed to improve SDM in particular, for example, by providing different scenarios or enabling input of patient preferences. Another potential limitation is that participation in the trial may influence physicians to talk about treatment options after graft loss, which may reduce the potential effect size of the AI intervention. Furthermore, the open-label design, which is unavoidable, introduces the possibility of bias, especially since the primary end point is not a medical end point.

### Conclusions

In conclusion, this study will generate novel and important data about the impact of AI on physician-patient interaction and SDM in the context of kidney transplantation, which may be applicable to other disease contexts as well.

## Appendix

Multimedia Appendix 1 Peer review report from the German Federal Ministry of Education and Research.

## Abbreviations

AE: adverse event
AI: artificial intelligence
AR: adverse reaction
CKD-EPI: Chronic Kidney Disease Epidemiology Collaboration
CPS: Control Preference Scale
DSS: decision-support system
eGFR: estimated glomerular filtration rate
KTC: kidney transplant center
PRIMA-AI: Prospectively investigating the Impact of AI on Shared Decision-Making in Post-Kidney Transplant Care
SDM: shared decision-making

## Data Availability

The data sets generated during and analyzed during this study are available from the corresponding author on reasonable request.
