# Supplementary material for: Investigating the Impact of AI on Shared Decision-Making in Post-Kidney Transplant Care (PRIMA-AI): Protocol for a Randomized Controlled Trial
Source: JMIR Res Protoc. 2024 Apr 1;13:e54857. doi: 10.2196/54857 (PMC11019425; doi:10.2196/54857)
Supplement: Multimedia Appendix 1 [file resprot_v13i1e54857_app1.pdf]

DLR Projektträger, Heinrich-Konen-Straße 1, 53227 Bonn

Universität Regensburg, Medizinische Soziologie
Frau Prof. Dr. Anne Herrmann-Johns
Universitätsstraße 31
93053 Regensburg

DLR Projektträger
Bereich Gesundheit

Ihre Ansprechpartner:
wissenschaftliche/r Mitarbeiter/in: Dr. Baddack-Werncke
Telefon +49 228 3821- 1692
E-Mail uta.baddack-werncke@dlr.de
Administrative/r Mitarbeiter/in: Anna Frants
Telefon +49 228 3821- 2579
E.Mail anna.frants@dlr.de
Fax +49 228 3821- -1257

Datum 14. März 2022

Bekanntmachung des Bundesministeriums für Bildung und Forschung „Förderung von Forschungsprojekten zu ethischen, rechtlichen und sozialen Aspekten in den Lebenswissenschaften" vom 19. Mai 2021

Ihre Projektskizze „ *PRospectively investigating the impact of AI on shared decision MAking in post-kidney transplant care"* vom 14.09.2021

Sehr geehrte Frau Professorin Dr. Herrmann-Johns,

vielen Dank für Ihre Beteiligung an der oben genannten Bekanntmachung des BMBF. Ihre oben genannte Projektskizze haben wir unter Einbeziehung eines interdisziplinär und international besetzten Begutachtungsgremiums eingehend geprüft.

Wir freuen uns Ihnen mitteilen zu können, dass Ihre Projektskizze **zur Förderung empfohlen** wird. In der Anlage finden Sie die detaillierte Bewertung Ihrer Skizze. Die Bewertung enthält weitere Empfehlungen und Hinweise.

Bitte reichen Sie nun die Formanträge ein. Bitte legen Sie mit den Formanträgen eine Stellungnahme zur Umsetzung der folgenden Auflagen und Empfehlungen vor:

- Bitte geben sie an, wie die Ergebnisse der Teilprojekte zusammenfließen oder sich gegenseitig informieren.
- Ziehen Sie in Erwägung relevante Akteure einzubinden.
- Eine Kooperation mit dem Projekt EPAMeDeM dieser Fördermaßnahme sollte angestrebt werden.

Ihren Formanträgen legen Sie bitte das in Ihrer Projektskizze dargestellte Finanzgerüst zugrunde. Das Finanzgerüst steht unter dem Vorbehalt der weiteren Prüfung der Zuwendungsfähigkeit.

Bitte verwenden Sie für die Erstellung der Formanträge das elektronische Online-Antragssystem **easy-Online** (https://foerderportal.bund.de/easyonline). Der direkte Link zu Ihrer Fördermaßnahme lautet: https://foerderportal.bund.de/easyonline/reflink.jsf?m=GP-ETHIK&b=ELSA-OPEN-2021 . Im „Formularschrank" des BMBF, der ebenfalls über das Förderportal des Bundes zu erreichen ist, finden Sie die **Richtlinien für Zuwendungsanträge auf Ausgabenbasis / Kostenbasis** sowie das

Merkblatt Vorkalkulationen für Zuwendungen - Kostenbasis -. Die Richtlinien sind bei der Erstellung des Formantrags und der Vorhabenbeschreibungen unbedingt zu beachten. Insbesondere ist die in den Richtlinien / im Merkblatt vorgegebene Gliederung der Vorhabenbeschreibung zu übernehmen.

Die Papierform der formgerechten Anträge muss rechtsverbindlich unterschrieben werden.

Wir bitten Sie als Verbundkoordinatorin, alle Formanträge Ihres Verbunds gebündelt an die folgende Postadresse zu senden:

DLR Projektträger
- Bereich Gesundheit -
Dr. Uta Baddack-Werncke / Anna Frants
Heinrich-Konen-Straße 1
53227 Bonn

Wenn Ihre Formanträge bis zum **01.05.2022** vollständig vorliegen, kann die Laufzeit der Verbundvorhaben voraussichtlich zum **01.09.2022** beginnen. Sollten Sie den vorgeschlagenen Einreichungstermin nicht einhalten oder sollten uns die Antragsunterlagen bis zu diesem Zeitpunkt nicht vollständig vorliegen, werden wir den Förderbeginn des Verbunds verschieben. Eine rückwirkende Bewilligung ist aus zuwendungsrechtlichen Gründen nicht möglich.

Wir weisen Sie darauf hin, dass alle obigen Angaben eine Zwischenmitteilung zum derzeitigen Stand unserer Prüfung Ihrer Projektskizze darstellen. Die abschließende Prüfung wird erst nach der Vorlage des vollständigen Formantrags möglich sein.

Sie können aus diesem Schreiben **keinesfalls** einen Rechtsanspruch auf Förderung ableiten. Insbesondere können Sie bei Ablehnung Ihres Formantrags keinen Ersatz für angefallene Ausgaben bzw. Kosten erhalten.

Bitte teilen Sie dieses Zwischenergebnis umgehend Ihren Verbundpartnern mit. Wir werden Ihnen hierzu dieses Schreiben parallel auch per E-Mail schicken.

Sollten Sie Fragen haben, rufen Sie uns gerne an.

Wir wünschen Ihnen viel Erfolg und freuen uns auf eine gute Zusammenarbeit.

Mit freundlichen Grüßen

i. A.   i. A.

Baddack

Dr. Baddack-Werncke   Anna Frants

Anlagen
- Detaillierte Bewertung der Projektskizze
- Empfohlenes Finanzgerüst für die Formantragstellung
- Vorlage Datenmanagementplan
- Hinweise und Checkliste zur Formantragstellung
- Muster ressourcenbezogener Arbeitsplan

Detaillierte Bewertung der Projektskizze

**Proposal No.:**	
**Title:**	PRospectively investigating the impact of AI on shared decision MAking in post-kidney transplant care
**Project Investigator:**	**Prof. Dr. Anne Herrmann-Johns**
**Institution:**	**Universität Regensburg, Medizinische Soziologie**

Evaluation:

The proposal aims to examine the impact of AI in shared decision making in the healthcare setting.
The proposal has good potential to address an important gap in the literature: empirical understanding of uses of AI in SDM from multiple actors/ perspectives (within and beyond the kidney transplantation usecase) and to ultimately contribute to improvements in healthcare through improved information and decision support.
The applicants comprise a very strong team, each with deep understanding and experience of the project proposed. The interdisciplinary approach and the expertise of the involved researchers are convincing.
Minor weakness: the transition of the empirical to ethical conclusions not clearly specified.
Recommendation:
• Clearly state how the findings of the subprojects will be synthesized or will inform one another.
• Consider co-production/ involvement with stakeholders.
• The project should consider cooperation with the EPAmeDem team.

Anlage – Empfohlenes Finanzgerüst

„PRospectively investigating the impact of AI on shared decision MAking in post-kidney transplant care “

Für die Formanträge Ihres Verbundes werden die folgenden Finanzgerüste zur Beantragung empfohlen. Die Finanzgerüste stehen unter dem Vorbehalt der weiteren Prüfung der Zuwendungsfähigkeit.

Hochschulen und Universitätskliniken können darüber hinaus eine Projektpauschale in Höhe von 20 Prozent der zuwendungsfähigen Gesamtausgaben beantragen. Die Beantragung erfolgt in diesem Fall mit dem Formantrag AZAP.

Antragstellender: Universität Regensburg
Teilprojekt: Herrman-Johns (1)

AZA Position	Inhalt	Euro
0812	Wissenschaftliche MA*	
0822	SHK, administrativer Support	
0835	Vergabe von Aufträgen	
0843	allgemeine Verwaltungsausgaben (Open access Publikationen [0841], Veranstaltungsorganisation [0841], Rekorder, Laptops**)	
0846	Reisemittel	
PP	20%	
Gesamt		376.669,93

Antragstellender: Universität Erlangen-Nürnberg
Teilprojekt: Dabrock (2)

AZA Position	Inhalt	Euro
0812	Wissenschaftliche MA*	
0822	SHK	
0843	allgemeine Verwaltungsausgaben (Open access Publikationen [0841], Veranstaltungsorganisation [0841])	
0846	Reisemittel	
PP	20%	
Gesamt		206.047

Antragstellender: DFKI GmbH Berlin
Teilprojekt: Möller (3)

AZK Position	Inhalt	Euro
0813	Verbrauchsmaterial (Open access Publikationen)	
0837	SHK, administativer Support, PhD Stelle*	
0838	Reisemittel	
0860	Verwaltungsausgaben	
Gesamt		201.174,37

Antragstellender: Charité Universitätsmedizin
Teilprojekt: Budde (4)

AZA Position	Inhalt	Euro
0812	Wissenschaftliche MA*	
0822	SHK	
0846	Reisemittel	
PP	20%	
Gesamt		181.010,16

Anmerkung:

* Sofern das Personal bereits bekannt ist, bitten wir Sie, die personenbezogene Gehaltsrechnung (Aufschlüsselung nach Arbeitgeberanteilen) als Tabelle dem Antrag beizufügen. Bitte geben Sie an, nach welchem Tarif und in welcher Stufe das Personal vergütet wird. Bei noch ungekanntem (N.N.) Personal ist entsprechend der Qualifikation maximal die Vergütung nach E 13 Stufe 2 zulässig. Ein Stufenaufstieg ist bei unbekanntem Personal nicht möglich.

**Bitte grenzen Sie die anzuschaffenden Laptops von der Grundausstattung ab.
